# CD11c+ M1-like macrophages (MΦs) but not CD206+ M2-like MΦ are involved in folliculogenesis in mice ovary

**DOI:** 10.1038/s41598-018-25837-3

**Published:** 2018-05-25

**Authors:** Yosuke Ono, Miwako Nagai, Osamu Yoshino, Kaori Koga, Allah Nawaz, Hideki Hatta, Hirofumi Nishizono, Gentaro Izumi, Akitoshi Nakashima, Johji Imura, Kazuyuki Tobe, Tomoyuki Fujii, Yutaka Osuga, Shigeru Saito

**Affiliations:** 1 Department of Obstetrics and Gynecology, University of Toyama, Toyama, Japan; 2 Department of Obstetrics and Gynecology, University of Tokyo, Tokyo, Japan; 3 First Department of Internal Medicine, University of Toyama, Toyama, Japan; 4 Department of Diagnostic Pathology, Graduate School of Medicine and Pharmaceutical Sciences, University of Toyama, Toyama, Japan; 5 Division of Animal Resources and Development, University of Toyama, Toyama, Japan

## Abstract

Macrophages (MΦs) are involved in folliculogenesis and ovulation. However, it is unknown which type of MΦ, M1 or M2, plays a more essential role in the ovary. CD206 or CD11c diphtheria toxin receptor transgenic (DTR) mice, which enable depletion of CD206+ M2 MΦs and CD11c+ MΦ or CD11c+ Dendritic cells (DCs), respectively, were used. Oocytes were used for *in vitro* fertilization and embryo transfer. *In vitro* fertilized embryos derived from M2 MΦ depleted oocytes were transferred to pseudo pregnant wild type mice. CD11c DTR mice were also used to investigate the role of CD11c cells, M1 MΦ and DCs in folliculogenesis. In WT mice, the proportion of CD206+ M2-like MΦs was not increased in follicular induction, while that of CD11c+ M1-like MΦs was increased. In CD206 DTR mice, folliculogenesis was normal and the ovulation number, fertilization rate, and implantation rate were similar to those in WT mice. In CD11c DTR mice, folliculogenesis was impaired with ovarian hemorrhage and the staining of platelet derived growth factor-receptor β (PDGF-Rβ), a marker of pericytes, and CD34, a marker of endothelial cells, was reduced. CD11c+ cells, M1 MΦs or DCs, may be involved in folliculogenesis, while M2 MΦs are not involved in folliculogenesis.

## Introduction

Macrophages (MΦs) are immune cells derived from bone-marrow precursors, and the differentiation of MΦs occurs in response to the surrounding cytokine milieu for acquisition of tissue-specific phenotypes^1^. MΦs contribute to the regulation of the pituitary-gonadal axis and are found throughout female reproductive tissues, including the ovary, uterus, oviduct, and mammary gland^2^. In the ovary, MΦs are the most abundant immune cells and are localized to thecal, luteal and interstitial tissue compartments and in the atretic follicle in both mouse and human^3–7^. MΦs have been shown to play diverse roles in ovarian events, such as follicular growth, ovulation and luteinization^8–16^. During follicular growth, it has been reported that the distribution of ovarian MΦs and the number of MΦs increase^17^. Some factors derived from MΦs such as, hepatocyte growth factor, epidermal growth factor and basic fibroblast factor, are known to influence follicular growth^18,19^. To elucidate the role of MΦs, some MΦs ablation methods have been performed. Van der Hoek *et al*. reported that administration of clodronate liposomes resulted in partial depletion of ovarian MΦs, leading to inhibition of follicle development and a significant decrease in ovulation rate^17^. The osteopetrotic mouse (op/op), in which the number of mature MΦs is severely reduced due to a mutation in the colony stimulating factor-1 (CSF-1) gene, showed a significant decrease in the number of growing follicles^20^. The CD11b diphtheria toxin-receptor (DTR) transgenic mouse model, a novel method of pan-MΦs ablation, has been used for various disease studies to investigate the role of MΦs^21–23^. In this mouse model, diphtheria toxin (DT) administration results in rapid and near complete ablation of pan-MΦs. Using CD11b DTR mice, Turner *et al*. demonstrated that pan-MΦ ablation during folliculogenesis resulted in ovarian hemorrhage with endothelial cell depletion and follicular atresia^24^. These hemorrhages were not observed in other tissues, suggesting that MΦs play a critical role in maintaining ovarian vascular integration during folliculogenesis. MΦs have been classified into two groups, includingM1 MΦs, which are classically activated MΦs with inflammatory effects, and M2 MΦs, that are alternatively activated MΦs with anti-inflammatory and remodeling effects^25^. However, it is not clear which subtype of MΦ is involved in folliculogenesis. As CD206 is a M2 MΦ specific marker, CD206 DTR mice, which was recently established in our institute^26,27^, would be useful to investigate the role of M2 MΦs in folliculogenesis. In the present study, using CD206 DTR mice, we examined the role of M2 MΦs in folliculogenesis, ovulation, and luteinization, as well as the impact on fertilization and implantation potential of oocytes derived from M2 MΦ depleted mice. During folliculogenesis, CD11b DTR, a pan-MΦ depleted mouse, exhibited follicle atresia with bleeding^24^. Through the accumulation of knowledge obtained from the CD206+ M2 MΦ and CD11c+ M1 MΦ and DC depletion models, and by comparing these data to CD11b DTR mouse data, we further investigated the role of MΦs in folliculogenesis.

## Methods

### Animal models and treatments

Female, CD11c DTR^28^ and CD206 DTR mice^26,27^, from 4 to-12-weeks-old, excluding 6 to 8 week-old mice, in which ovulatory number is unstable due to the effect of first wave of ovulation^29^, were used. For mice aged 9 to 12 weeks, we confirmed the regular estrus cycle via vaginal smear and housed in a specific pathogen free (SPF) animal facility with a controlled environment, 22–24 °C and 60–70% relative humidity, and on a light/dark cycle (12 h light/12 h dark) with food and water ad libitum. All animal experiments were performed according to the protocol approved by the Animal Care and Use Committee of University of Toyama and University of Tokyo.

### Flow cytometry

**Figure 1:**
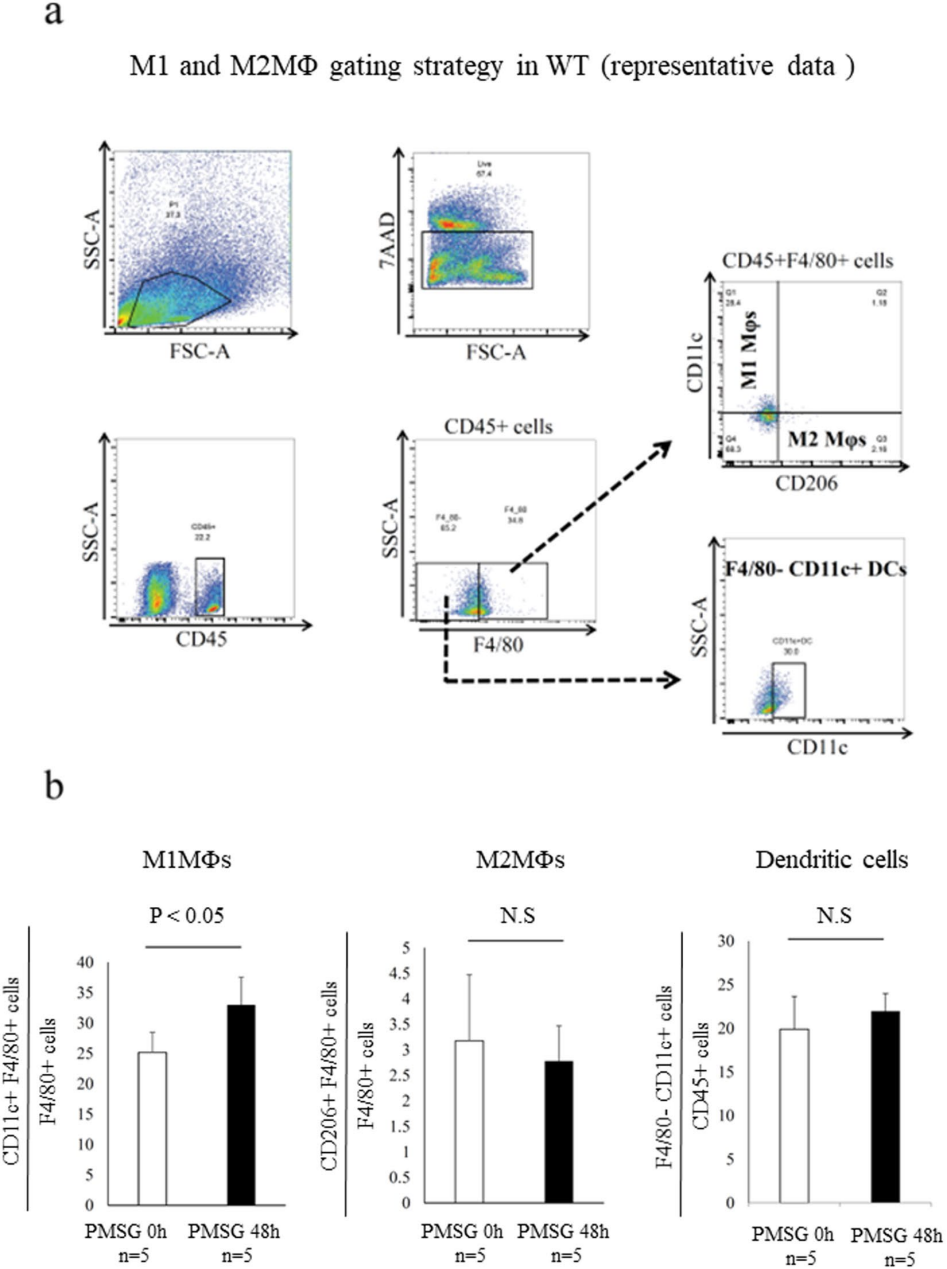
M1 and M2 macrophages (MΦs) in wild type (WT) mice ovary. (**a)** Representative flow cytometry analysis of M1 and M2 MΦs in the WT mice ovary. M1-like MΦ was defined as CD45+/F4/80+/CD11c+/CD206− cells, and M2-like MΦ was defined as CD45+/F4/80+/CD206+/CD11c− cells and Dendritic cells (DCs) was defined as F4/80− CD11c+ cells. (**b)** The proportion of CD11c+F4/80+ cells, M1-like MΦs (left panel) and CD206+ F4/80 cells, M2-like MΦs (middle panel) and F4/80− CD11c+ cells, DCs (right panel) in ovary. The proportion of M1-like MΦs significantly increased following follicular induction with PMSG 48 h, while that of M2-like MΦs and DCs was not increased. The data are shown as the means ± standard error of the mean (SEM). A *P*-value of < 0.05 was considered statistically significant by *Mann-Whitney U test*. N.S; not significant compared to WT mice. n; the number of mice.

Isolation and separation of the ovary and subsequent flow cytometry were performed as previously described^30^. Hamster anti-mouse CD11c monoclonal conjugated with PE (Cat# 553802), and 7-amino-actinomycin D [7AAD] (Cat# 559925) were obtained from BD Biosciences (Tokyo, Japan). The rat monoclonal antibody for anti-mouse CD206 conjugated with alexa fluor 647 (MCA2235A647) and the rat IgG2a conjugated with alexa flour 647 isotype antibody (Cat# 1212A647) were obtained from AbD Serotec Co. (Oxford, UK). In ovarian cells, after exclusion of dead cells by gating with 7-amino-actinomycin D, live cells were used for further analysis. M1 or M2 MΦs were identified as CD45+/F4/80+/CD11c+/CD206− or CD45+/F4/80+/CD206+/CD11c− cells, respectively (Fig. 1a). DCs were identified as CD45+/F4/80−/CD11c+ cells (Fig. 1a). These experiments were performed with a FACS Diva Version 6.1.2 automated cells analyzer (Becton Dickinson FACS Canto II). Data analyses were performed using Flow Jo software. Unstained specimen and isotype negative control were used for all relevant samples to justify gating strategy. Fluorescence minus one (FMO) control was used wherever needed.

### Immunohistochemistry

Paraffin-embedded tissues were cut 5-μm thick and mounted on slides. Ovarian sections of wild type, CD11c DTR and CD206 DTR mice were de-paraffinized in xylene, rehydrated through a graded series of ethanol, and washed in water. Antigen retrieval was performed in 10 mM sodium citrate buffer (pH 6.0) by microwaving for 10 min and then cooling to room temperature. Slide staining with the first and second antibodies was performed according to the manufacturer’s instructions. The immunostaining was performed in formalin-fixed, paraffin-embedded sections using specific antibodies to Ki-67 (Abcam, Tokyo, Japan, Cat# 15580, 1:100 dilution), Platelet derived growth factor-receptor β (PDGF-Rβ) (GeneTex Cat# 83371, 1:100 dilution), PDGF-B (Abcam Cat# 23914, 1:250 dilution), and CD34 (Abcam Cat# 81289, 1:50 dilution). For frozen sections, ovaries from mice were collected in 4% paraformaldehyde after systemic perfusion. The tissues were kept at room temperature for 2–3 h. Next, tissues were incubated in sterile phosphate buffered saline (PBS) for one overnight and 30% sucrose for one overnight in a shaker at 4 °C. Finally, the tissues were placed in blocks by adding OCT compound (Sakura Finetek, Tokyo, Japan) and the blocks were immediately stored at −80 °C for at least 24 h to solidify. Then the frozen tissues were cut into 10μm sections using a cryostat. After making the frozen block, immunofluorescence staining was performed using anti-rat CD206 (AbD Cat# MCA2235F, 1:50 dilution). All micrographs were taken with Keyence BZ-8000, TCS SP5 Leica confocal microscopes (Leica Microsystems K.K, Tokyo, Japan, Oil 63×).

### Reverse transcription (RT) and quantitative real-time polymerase chain reaction (PCR) analysis

Total RNA was extracted from mouse tissues, using the ISOGEN- II (NIPPON GENE Co. Tokyo, Japan). RT was performed using Rever Tra Ace qPCR RT Master Mix with gDNA Remover (TOYOBO Co. Tokyo, Japan). About 0.5–1 μg of total RNA was reverse-transcribed in a 20-μL volume. For the quantification of various mRNA levels, real time PCR was performed using the Mx3000P Real-time PCR System (Agilent Technologies, CA, USA) according to the manufacturer’s instructions. The PCR primers used with the SYBR Green methods were selected from different exons of the corresponding genes to discriminate PCR products that might arise from possible chromosomal DNA contaminants. The SYBR Green thermal cycling conditions were 1 cycle of 95 °C for 30 s, and cycles of 95 °C for 10 s, 60 °C for 10 s and 72 °C 10 s. The relative mRNA levels were calculated using the standard curve method and were normalized to the mRNA levels of GAPDH (forward, 5′-AATGTGTCCGTCGTGGATCTGA-3′ and reverse, GATGCCTGCTTCACCACCTTCT).

### Measurement of estradiol (E2) and progesterone (P4) levels

Mouse blood samples were collected when sacrificed. Serum levels of E2 and P4 were measured in duplicate, by EIA kits (Cayman Chemical, Michigan, USA).

### DT injection

DT was purchased from Sigma-Aldrich (St. Louis, MO, USA). DT was diluted with sterile PBS to the desired concentration and was intraperitoneally injected to mice. In CD11c DTR mice, DT was injected at a dose of 5 ng/gram body weight one time. In CD206 DTR mice, DT was injected at a dose of 30 ng/gram body weight three times every other day. The experiments and procedures were performed 48 h after the last injection. The depletion rates of CD11c+ cells and CD206+ cells in spleen were confirmed by flow cytometry analysis (Supplemental Fig. 1).

### Statistical analysis

Non-normally distributed data were analyzed by nonparametric tests (*Mann–Whitney U test*) using JMP software (SAS Institute Inc., Cary, NC, USA). A *P*-value of < 0.05 was considered statistically significant.

## Results

### Changes in the number of CD11c+ M1-like and CD206+ M2-like MΦs after 48 h PMSG treatment

**Figure 2:**
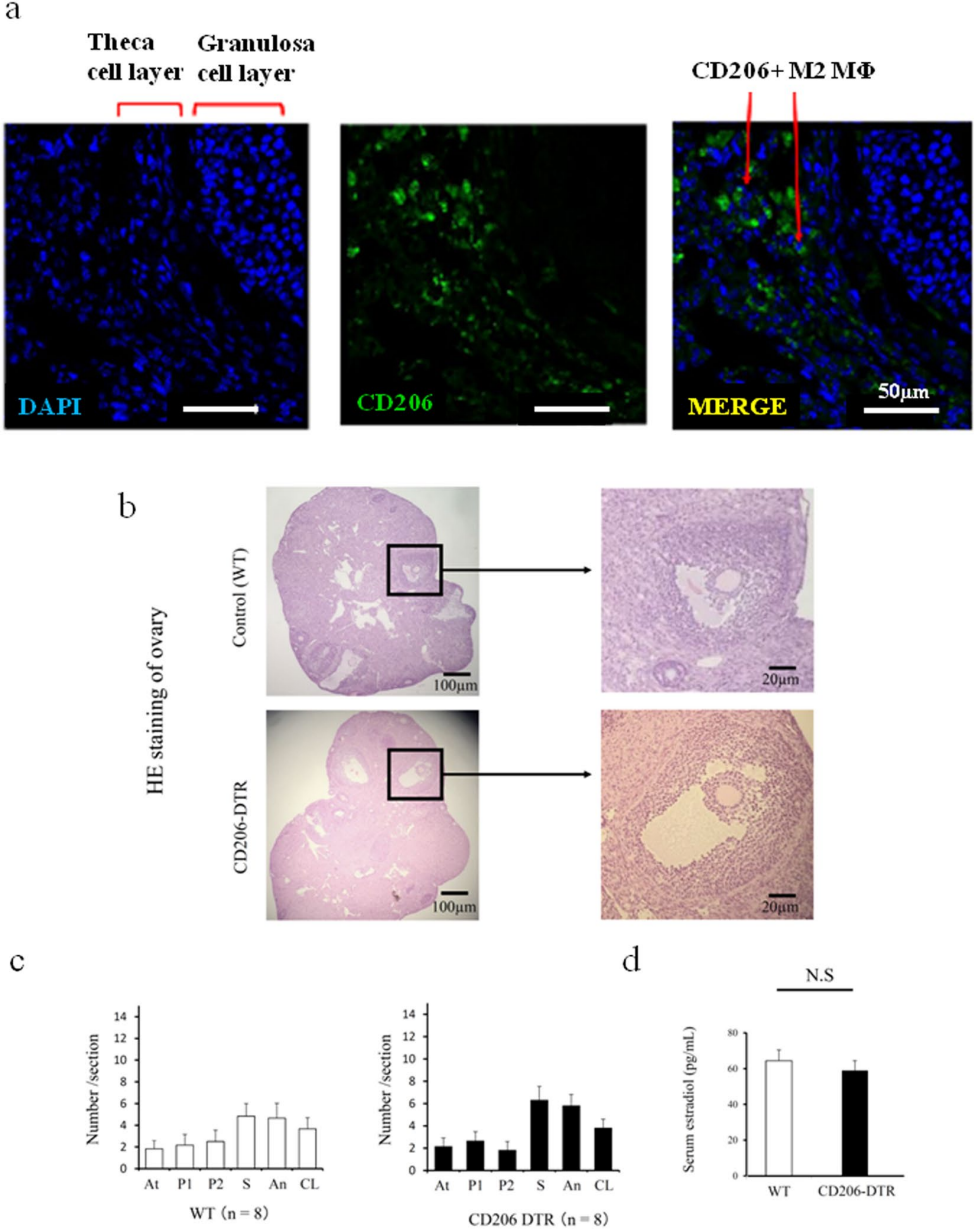
The involvement of CD206+ F4/80+ M2-like macrophage (MΦ) in mouse ovary during folliculogenesis. (**a)** Localization of ovarian CD206+ M2-like MΦ in follicular induction with PMSG 48 h. (**b)** The Hematoxylin Eosin (HE) staining of ovaries in follicular induction with PMSG 48 h in wild type (WT) and CD206 Diphtheria toxin-receptor (DTR) mice. (**c)** The number of each follicle stage in follicular induction in WT and CD206 DTR mice. We counted the number of follicles at each stage, including atresia (At), primordial (P1), primary (P2), secondary (S), antral (An), and corpus luteum (CL) in CD206 DTR (right panel) and WT (left panel) mice, as previous described^32^. (**d)** The serum estradiol levels after 48 h PMSG in WT and CD206 DTR mice.n; the number of mice. N.S; not significant compared to WT mice.

Folliculogenesis was induced with pregnant mare serum gonadotropin (PMSG, Sigma Aldrich) 10 IU for 48 h (Supplemental Fig. 2)^31^. In wild type mice (WT) ovary, M1 and M2 MΦs were detected by flow cytometry (Fig. 1a) and flow cytometry analysis revealed that the proportion of CD11c+ F4/80+ M1-like MΦs was significantly increased in the ovary (*P* < 0.05, Fig. 1b, left panel), while the proportions of CD206+ F4/80+ M2-like MΦs (Fig. 1b, middle panel) and CD11c+ F4/80-DCs (Fig. b, right panel) were not increased. Using wild type mice, in the ovary treated with PMSG (10IU) for 48 h, CD206+ MΦs were located mainly in the theca cell layer (Fig. 2a). In CD206 DTR mice treated with PMSG for 48 h (Supplemental Fig. 2), in which M2 MΦs were depleted, the morphology of ovary was not changed compared to WT (Fig. 2b). Additionally, we counted the number of follicles at each stage, including atresia, primordial, primary, secondary, antral, and corpus luteum, in CD206-depleted and WT mice, as previously described^32^ (Supplemental Fig. 3). The numbers of each stage of follicle and the serum estradiol (E2) levels in CD206 depleted mice were comparable to WT (Fig. 2c and d, respectively).

### The association of CD206+ M2-like MΦs with ovulation or luteinization

**Figure 3:**
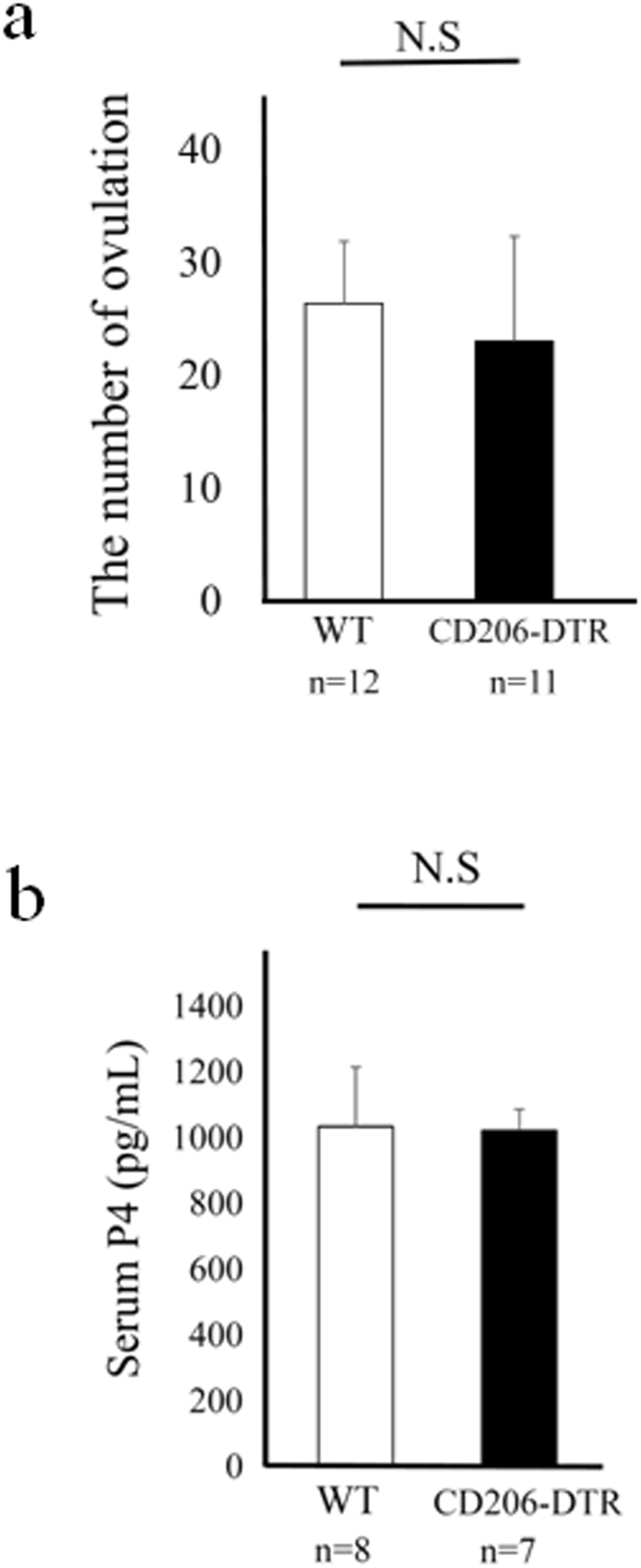
The involvement of CD206+ F4/80+ M2-like macrophage (MΦ) in mouse ovary during ovulation and luteinization. (**a)** The number of ovulations in wild type (WT) and CD206 diphtheria toxin-receptor (DTR) mice treated with superovulation. (**b)** Serum Progesterone (P4) levels in WT and CD206 DTR mice in ovulatory induction. n; the number of mice. N.S; not significant compared to WT mice.

In CD206 DTR mice, after superovulation with PMSG (10 IU) for 48 h followed by human chorionic gonadotropin (hCG) (10IU) for 15 h (Supplemental Fig. 4), the number of oocytes obtained from the fallopian tubes, and the serum progesterone (P4) levels were not changed compared to those of WT (Fig. 3a and b), suggesting that in addition to folliculogenesis, M2 MΦs were not involved in ovulation and luteinization.

### The impact of oocytes from CD206+ M2-like MΦ-depleted mice on fertilization and implantation

**Figure 4:**
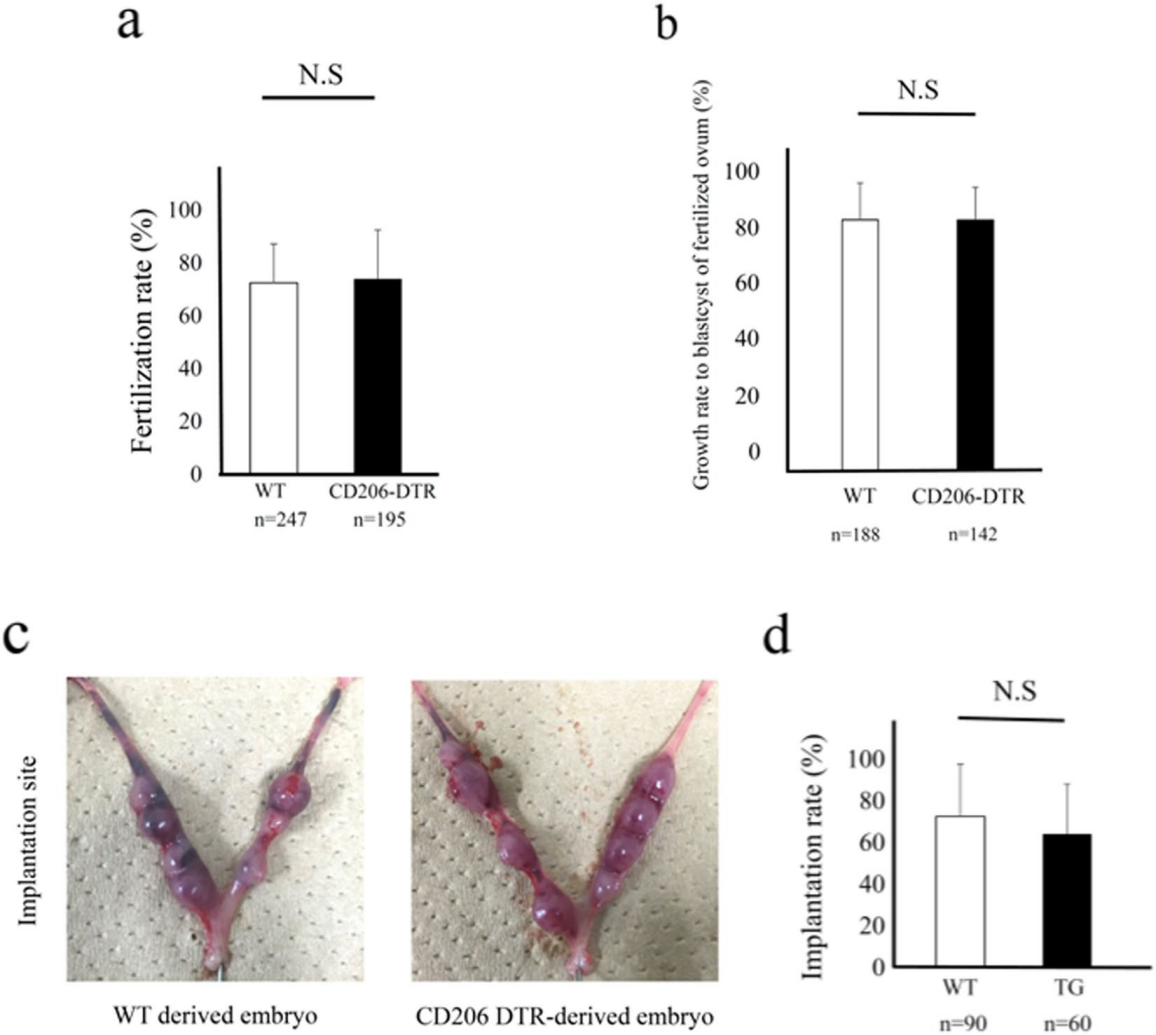
The involvement of CD206+ M2-like MΦs in fertilization and implantation. (**a)** *In vitro* fertilization rate of oocytes derived from wild type (WT) and CD206 diphtheria toxin receptor (DTR) mice. n; the number of oocytes. (**b)** The embryo growth rate to blastocyst of fertilized ovum derived from WT and CD206 DTR mice. (**c)** The photographs of implantation site after embryo transfer derived from WT mice (left panel) and CD206 DTR mice (right panel). (**d)** The implantation rate of ovum derived from CD206 DTR mice compared to WT mice. n; the number of embryo. N.S; significant compared to WT mice.

After superovulation, oocytes obtained from the fallopian tubes were used for *in vitro* fertilization (IVF) (Supplemental Fig. 5). The fertilization rate of CD206 DTR-mice derived oocytes was not changed compared to that of WT-mice derived oocytes (Fig. 4a). The growth rate to blastocyst of fertilized ovum derived from CD206 DTR mice was not changed compared to that of WT-derived oocytes (Fig. 4b). *In vitro* fertilized embryos from WT or CD206 DTR mice were used for the study for implantation. Ten embryos each were transferred to pseudo pregnant WT mice (n = 5, Supplemental Fig. 5). Using oocytes derived from CD206 DTR mice, the implantation rate was not changed compared to WT derived oocytes (Fig. 4c and d), suggesting that oocytes derived from M2-like MΦ-depleted mice had no effect on fertilization and implantation.

### The role of CD11c+ cells, M1-like MΦs and DCs in folliculogenesis

**Figure 5:**
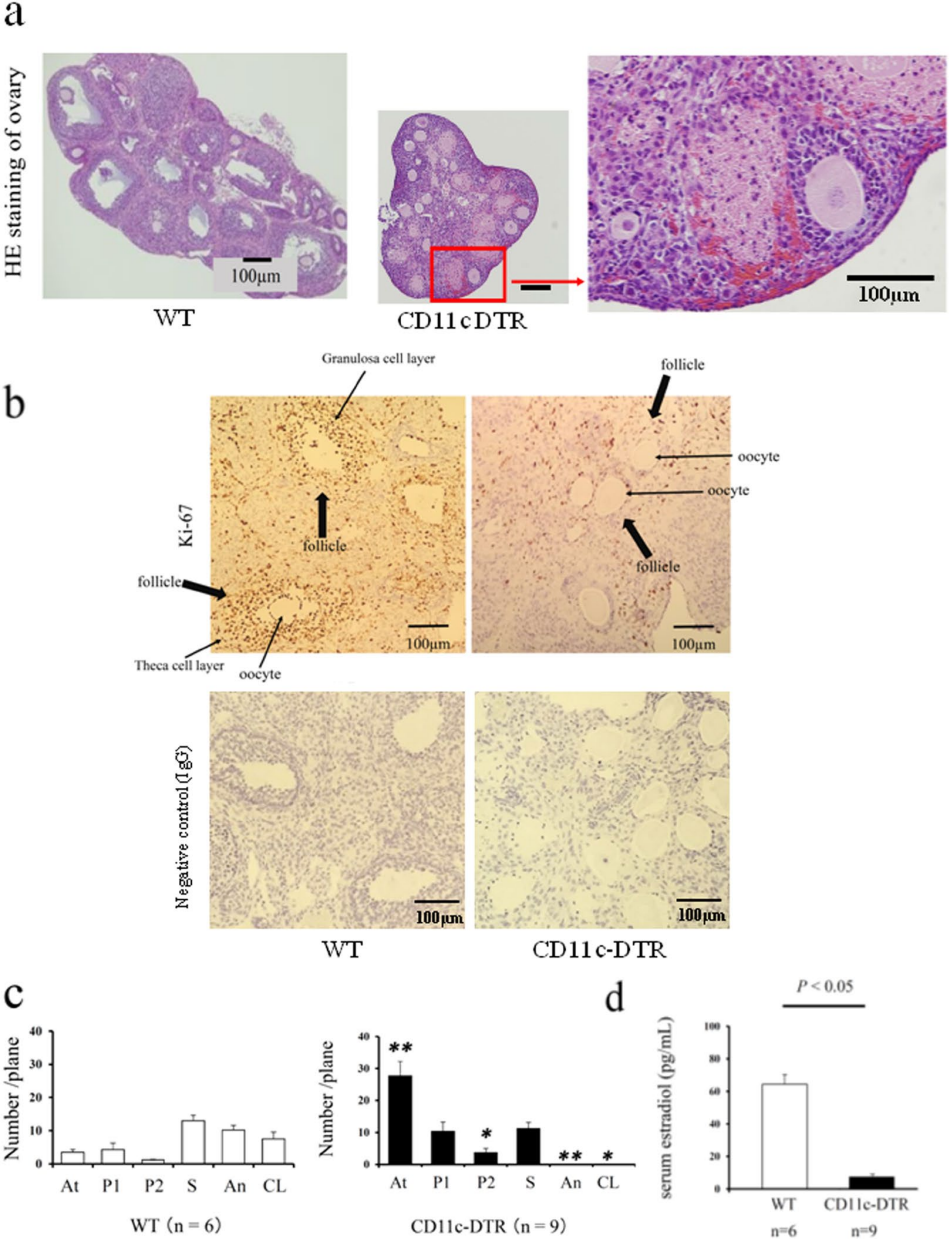
The involvement of CD11c+ cells, M1 macrophages (MΦs) and dendritic cells (DCs) in folliculogenesis. **(a)** The Hematoxylin Eosin (HE) staining of ovaries in follicular induction with PMSG 48 h in Wild Type (WT) (left Panel) and CD11c diphtheria toxin receptor (DTR) mice (middle). (**b)** The Ki-67 staining, a cell proliferation marker, in follicular induced ovary with PMSG 48 h in WT and CD11c DTR mice. Negative control data are also shown. (**c)** The number of each follicle stage in follicular induction in WT and CD11c DTR mice. We counted the number of follicles at each stage, such as atresia (At), primordial (P1), primary (P2), secondary (S), antral (An), and corpus luteum (CL) in CD206 DTR (right panel) and WT (left panel) mice, as previous described^32^. (**d)** The serum estradiol levels after 48 h PMSG in WT and CD11c DTR mice. The data are shown as the means ± SEM. A *P*-value of < 0.05 was considered statistically significant by *Mann–Whitney U test*. n; the number of mice. N.S: not significant compared to WT.

When CD11c+ cells were depleted by DT administration (Supplemental Fig. 1), the ovaries became atrophic with hemorrhage after PMSG stimulation for 48 h (Fig. 5a). Immunohistochemical staining for Ki-67 revealed that proliferating granulosa cells in CD11c+ cells depleted mice were very low compared to WT mice (Fig. 5b).

We counted the number of follicles at each stage in CD11c-depleted and WT mice. In CD11c-depleted mice, the numbers of atretic and primary follicles were significantly increased (*P* < 0.01, Fig. 5c), and no antral follicles were observed in the ovary.

These data indicated that in the absence of CD11c+ cells, antral follicles which produce E2 and require vascular network around follicles^33^ were severely impaired, resulting in atresia. In consistent with this notion, the serum E2 levels were very low compared to WT (*P* < 0.01, Fig. 5d).

**Figure 6:**
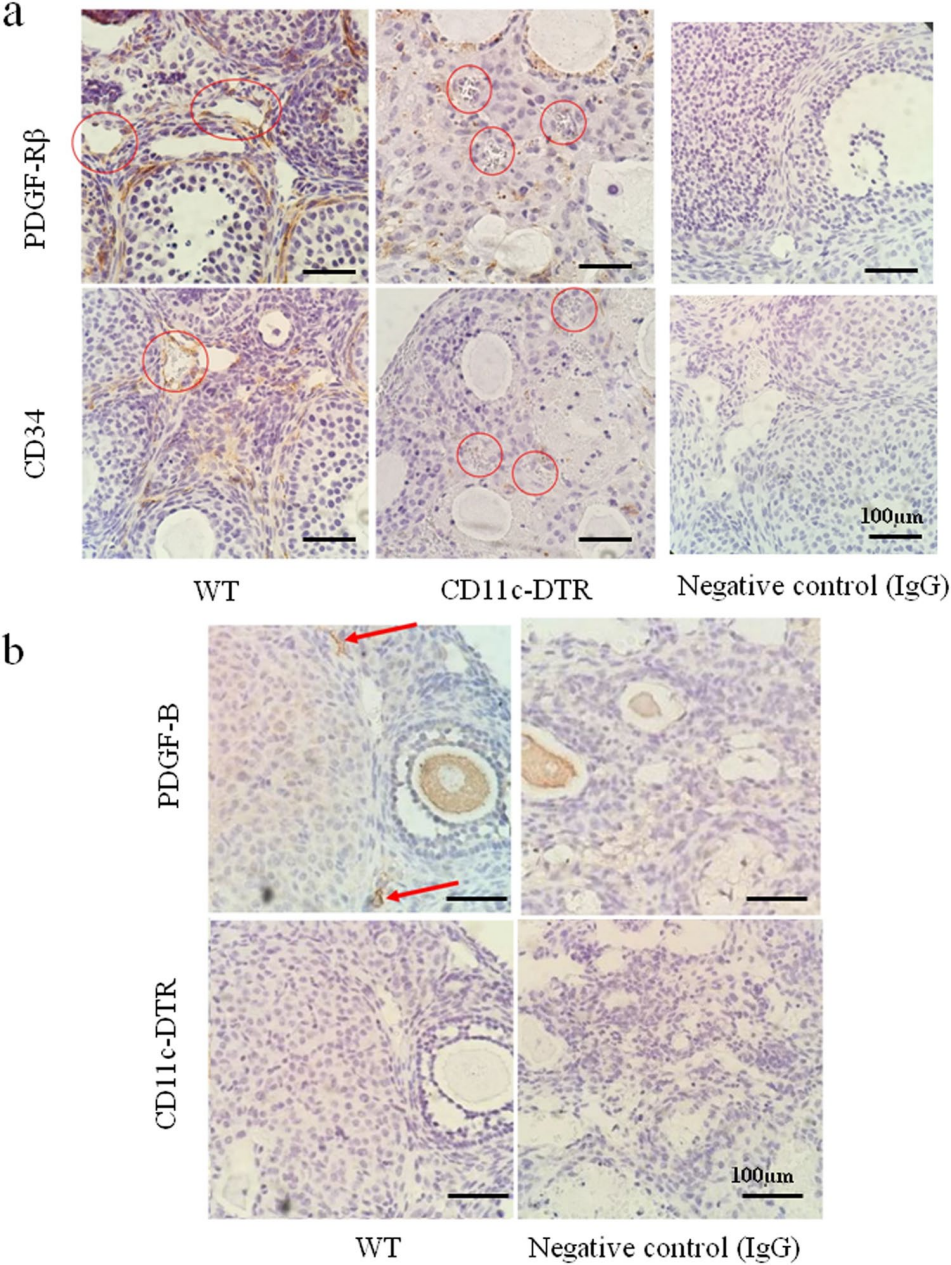
(**a**) Localization of PDGF-Rβ+ pericytes, and CD34+ endothelial cells, around follicles in WT and CD11c DTR mice. (**b**) Localization of PDGF-B around follicles in WT and CD11c DTR mice. Negative control data are shown.

To evaluate the mechanism of bleeding, immunostaining of PDGF-Rβ, a marker of pericytes, and CD34, a marker of endothelial cells, was performed. The numbers of PDGF-Rβ+ pericytes and CD34+ endothelial cells around follicles were decreased in CD11c DTR mice, but not in WT mice (Fig. 6a). PDGF-B, a ligand of PDGF-Rβ was positive at stromal lesions in WT mice (Fig. 6b, arrow), and negative in CD11c DTR mice.

## Discussion

Although MΦs are involved in various ovarian functions, such as folliculogenesis and ovulation^2,3,34^, the role of subset levels of MΦs, M1 or M2 MΦs, has not been reported. To further elucidate the role of MΦs, for the first time, we designed a comprehensive study to examine the roles of M2-like MΦs in the ovary using CD206 DTR mice.

In CD11b DTR mice, a pan MΦ depletion model, folliculogenesis was impaired, and defects in ovarian vasculogenesis, resulting in hemorrhage, were observed^24^. Therefore, we specifically focused on folliculogenesis in the subsequent study. We found that in CD206+ DTR mice, folliculogenesis was normal, so we speculated that M1 MΦs might be involved in folliculogenesis. As there is no specific depletion mouse model of M1 MΦs, we utilized the CD11c DTR mice, in which both M1-like MΦs and DCs can be depleted^35,36^. Using CD11c DTR mice, Cohen *et al*. found the depletion of CD11c+ cells during the ovulatory period resulted in anovulation due to impairment of cumulus expansion of granulosa cells, which is restored with transplantation of DC^37^. In the present study, we depleted CD11c+ cells at the time of folliculogenesis, not ovulation, and found that depletion of CD11c+ cells resulted in follicular atresia with hemorrhage, which is similar to the outcome observed in CD11b DTR mice^24^. In late phase of secondary follicles onwards, which are gonadotropin-dependent, a well-organized vascular network is essential so that gonadotropin and growth factors can reach the follicles^33^. In CD11c DTR mice, the proportion of antral follicles onward was severely decreased, suggesting that vascular network was impaired around follicles. There is growing evidence that the vascular network is formed by MΦs, pericytes, and endothelial cells^38,39^. Vascular endothelial growth factor (VEGF) is known to be an angiogenesis factor produced by M2MΦs^40^ and be involved in folliculogenesis in mice^41^. Also, high ratio of matrix metalloproteinase-9 (MMP-9)/tissue inhibitor of metalloproteinase (TIMP-1) is known as an angiogenic status regulated by M2MΦs^42^. Therefore, we examined the VEGF, MMP-9 and TIMP-1 mRNA expression in CD11c DTR mice ovary, in which M2MΦs might be dominant. The levels of VEGF mRNA was not changed compared to WT (Supplemental Fig. 6), and higher ratio of MMP-9/TIMP-1 was observed (Supplemental Fig. 6), suggesting that these angiogenic factors derived from M2MΦs were not impaired in CD11c DTR mice. MΦs also produce an angiogenic factor, PDGF-B, to recruit pericytes through PDGF-Rβ^43^, and recruited pericytes interact with endothelial cells to form vascular integrity^43^. Disruption of this interaction would culminate in widespread hemorrhages^24^. Hemorrhages were found in the ovaries of CD11b DTR mice, a pan MΦ ablation model, due to depletion of endothelial cells^24^. Moreover, Kuhnert *et al*. reported that the blockade of PDGF-Rβ by administration of its decoy receptor resulted in bleeding in the ovary^44^. PDGF-B mutant embryos also develop fatal hemorrhage just prior to birth^45^. In contrast, Di Pietro M *et al*. reported that local administration of PDGF-B improved follicular development and ovarian angiogenesis in a rat model^46^. Our present study demonstrated that, in the absence of CD11c+ cells, PDGF-B signal was negative in stromal lesions and the numbers of PDGF-Rβ+ pericytes and CD34+ vascular endothelial cells around follicles were decreased, which is also observed in CD11b DTR mice^24^. As CD11c is not only a M1 MΦ but also a DC marker^47^, we can’t distinguish which CD11c+ cells contributed to the phenotype. According to the microarray data available on line, MΦs produce five times more PDGF-B, than DCs (BioGPS, http://biogps.org/#goto=welcome). Moreover, M1 MΦs are known to produce significantly more PDGF-B than M2 MΦs^48^. Collectively, in CD11c DTR mice, the observed phenotype, an atrophic ovary with bleeding, may be at least partly attributed to M1 MΦs.

The limitation of this study was that we could not exclude the role of DCs during folliculogenesis. Further study is needed to determine whether transplantation of M1 MΦs may reverse the ovarian bleeding in the CD11c DTR model. Alternatively, regulation of polarization from M2 to M1 MΦs by granulocyte macrophage-colony-stimulating factor (GM-CSF) or palmitic acid^49,50^ may rescue the phenotype observed in CD11c DTR mice. A better understanding and the control of M1MΦs in the ovary could facilitate a new strategy to treat cases of impaired folliculogenesis.

## Electronic supplementary material

Supplemental data and materials
