# Supplementary material for: CD11c+ M1-like macrophages (MΦs) but not CD206+ M2-like MΦ are involved in folliculogenesis in mice ovary
Source: Sci Rep. 2018 May 25;8:8171. doi: 10.1038/s41598-018-25837-3 (PMC5970206; doi:10.1038/s41598-018-25837-3)
Supplement: Supplementary file 1 — Supplemental data and materials [file 41598_2018_25837_MOESM1_ESM.pdf]

CD11c+ M1-like macrophages (MΦs) but not CD206+ M2-like MΦ are involved in folliculogenesis in mice ovary

Yosuke Ono[1*], Miwako Nagai[2*], Osamu Yoshino[1], Kaori Koga[2], Allah Nawaz[3], Hideki Hatta[4], Hirofumi Nishizono[5], Gentaro Izumi[2], Akitoshi Nakashima[1], Johji Imura[4], Kazuyuki Tobe[3], Tomoyuki Fujii[2], Yutaka Osuga[2 *] and Shigeru Saito[1 *]

Supplemental Material

Supplemental Figure 1. Confirmation of depletion of CD206+ cells and CD11c+ cells in spleen by flow cytometry analysis.

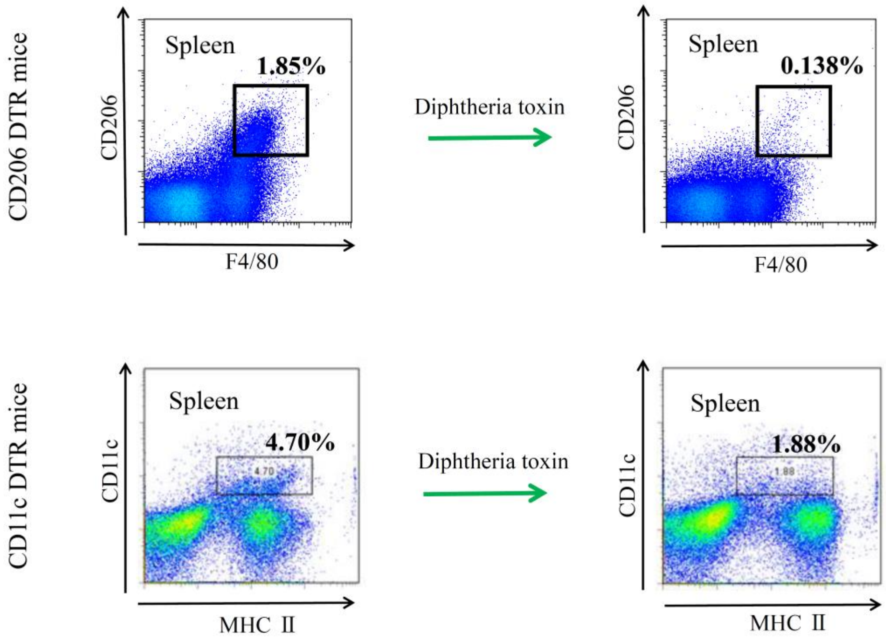

Supplemental Figure 2.

Follicular induction protocol in mice. Follicular growth was induced by PMSG 10 IU for 48h stimulation. Diphtheria toxin (DT) was used to deplete CD206+ and CD11c+ cells.

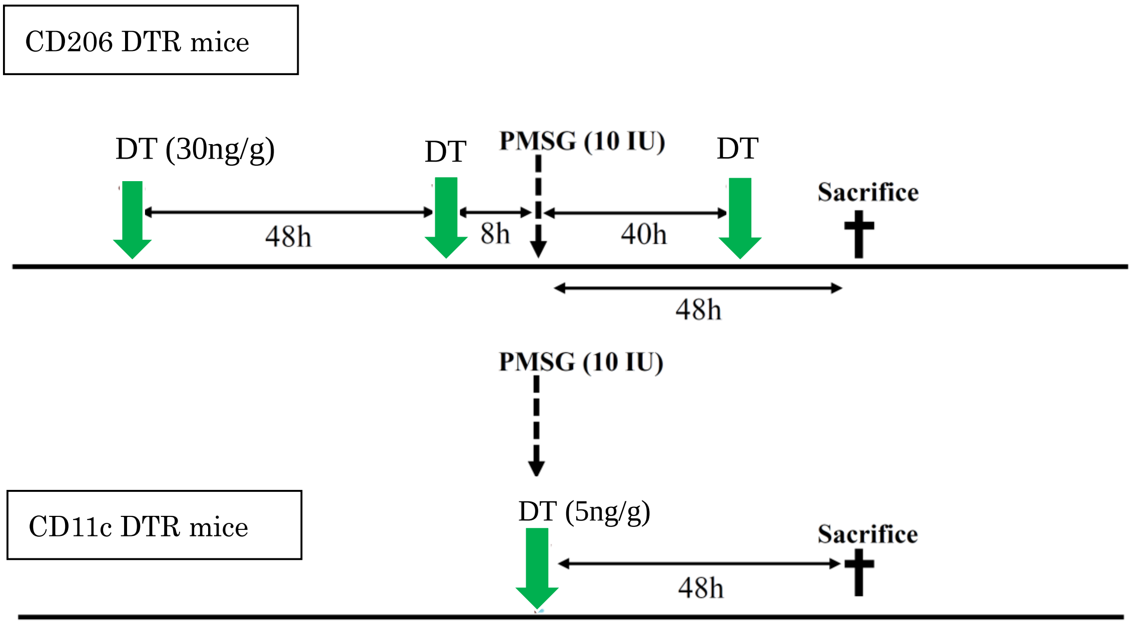

DTx： ：Diphtheria toxin

Supplemental Figure 3. How to count the number of follicles at each stage in wild type (WT) and CD11c-diphtheria toxin receptor (DTR) mice.

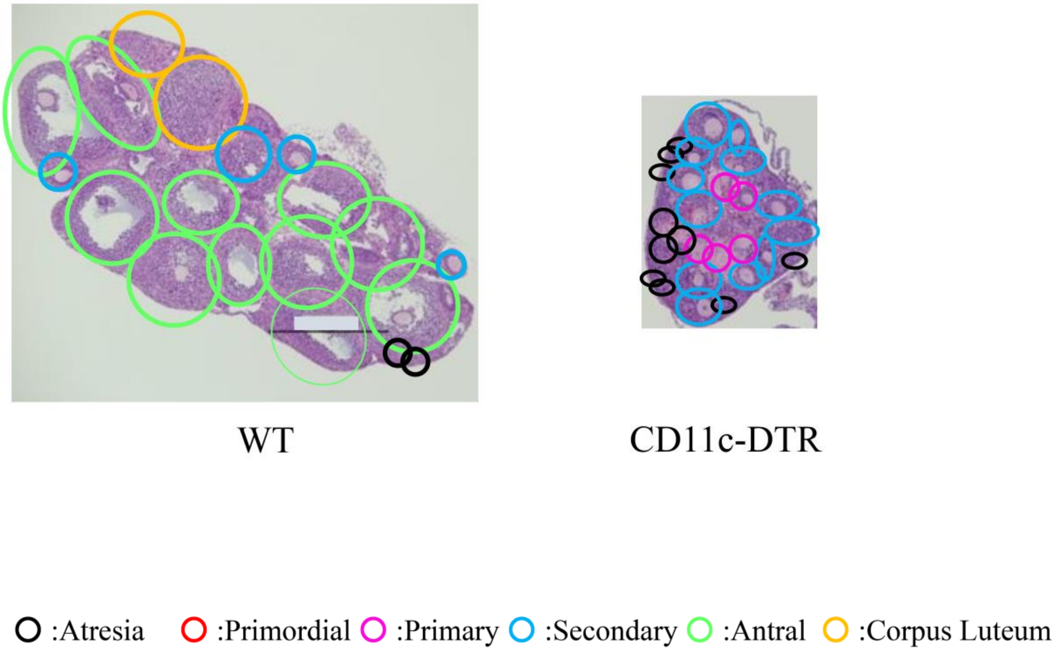

Supplemental Figure 4. The protocol to examine the role of M2 macrophages (MΦs) in ovulatory induction. Superovulation was induced by PMSG (10 IU) 48h followed by hCG (10IU) 15h. Diphtheria toxin (DT: 30ng/g) was used to deplete CD206+ cells.

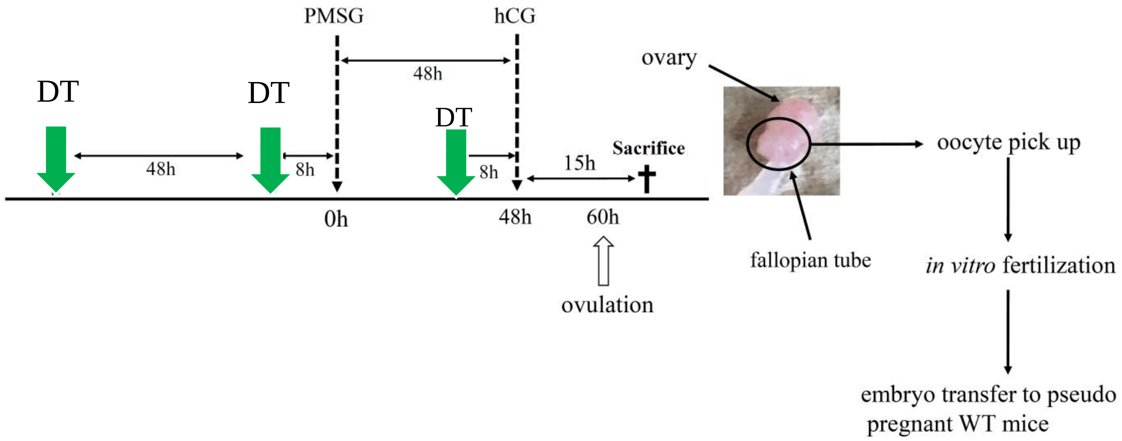

Supplemental Figure 5. The protocol to investigate the impact of oocytes derived from CD206 DTR mice for fertilization and implantation. WT and CD206 DTR mice derived oocytes were *in vitro* fertilized and these embryos were transferred to pseudo pregnant WT mice, respectively.

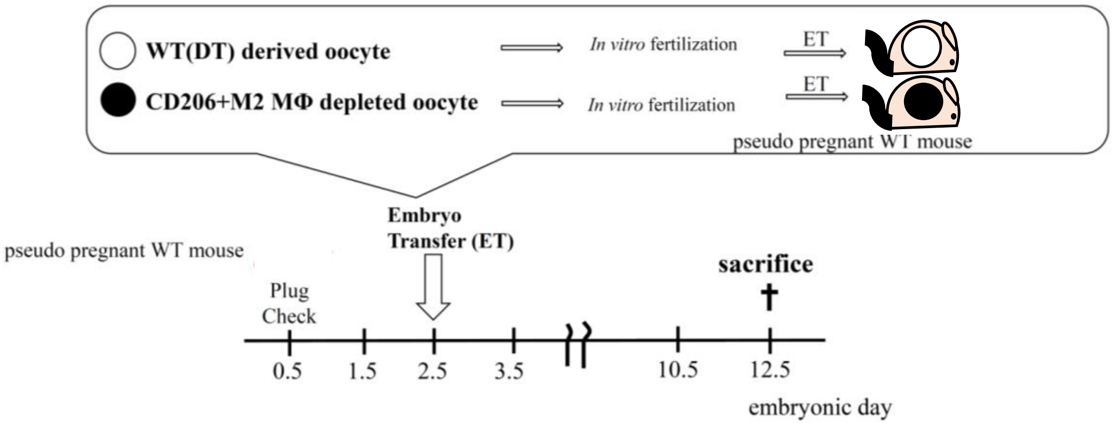

Supplemental Figure 6. Expressions of the mRNA of vascular endothelial growth factor (VEGF-A) and the ratio of matrix metalloproteinase-9 (MMP-9) / tissue Inhibitor of metalloproteinase (TIMP-1) in ovary in WT and CD11c DTR mice 48h after PMSG (10 IU) treatment by q-PCR.

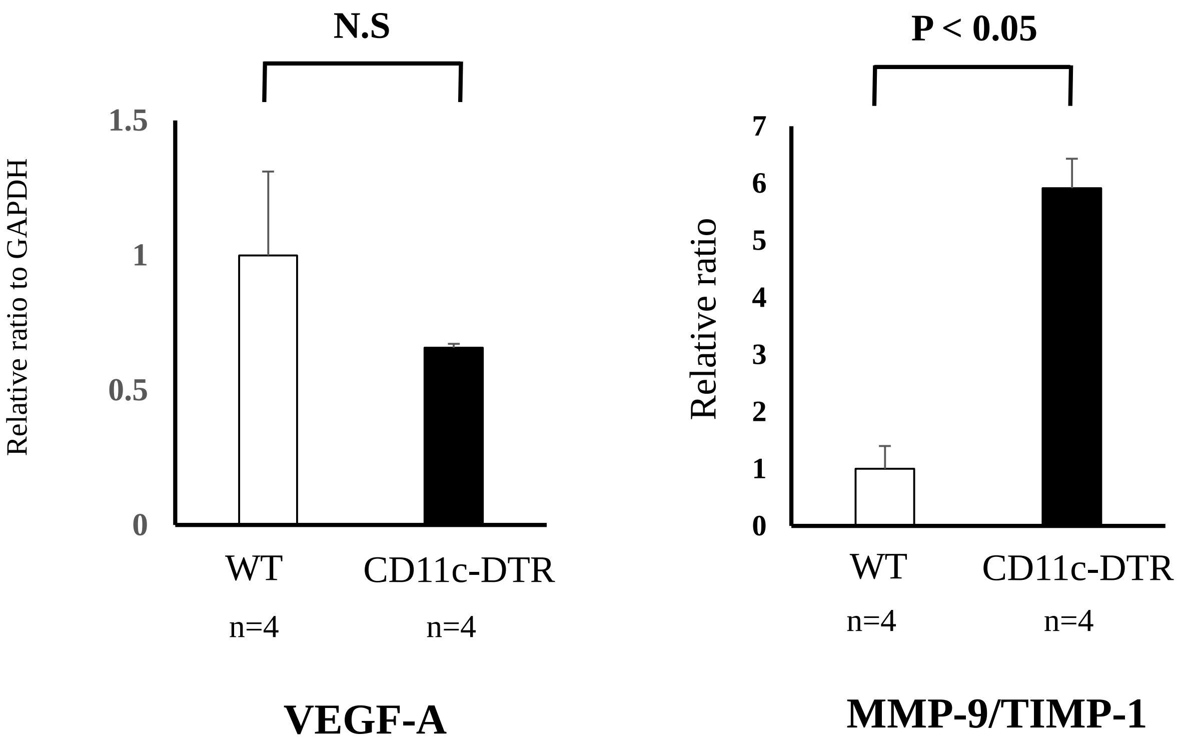

Primers sequences of MMP-9, VEGF are shown below

VEGF (forward, 5'-CCCACGACAGAAGGAGAGCAGAAGT- 3' and reverse, CATCAGCGGCACACAGGACGG). MMP-9 (forward, 5'

CGTCATTCGCGTGGATAAGG - 3’ and reverse, TTTGGAAACTCACACGCCAG).

TIMP-1 (forward, 5’-CCCTTTGCATCTCTGGCATC-3’ and reverse,

GCATTTCCCACAGCCTTGAA). The levels of VEGF mRNA was not changed compared to WT and higher ratio of MMP-9 / TIMP-1 was observed.

A *P*-value of < 0.05 was considered statistically significant by *Mann-Whitney U test*.

N.S; not significant compared to WT mice. n; the number of mice.
